# Identifying disruptors of male germ cell development by small molecule screening in *ex vivo* gonad cultures

**DOI:** 10.1186/1756-0500-6-168

**Published:** 2013-04-30

**Authors:** Stephanie I Wakeling, Denise C Miles, Patrick S Western

**Affiliations:** 1 Monash Institute of Medical Research, Monash University, Clayton, 27-31 Wright St, Clayton, VIC, 3168, Australia; 2 Current addresses: SIW: Deakin University, Geelong, VIC, 3220, Australia; 3 Department of Molecular Genetics, The Netherlands Cancer Institute, Amsterdam, the Netherlands

**Keywords:** Germ cell, Signaling, Testicular dysgenesis, Organ culture, Flow cytometry, Gonad, Germ cell tumour

## Abstract

**Background:**

Germ cell development involves formation of the spermatogenic or oogenic lineages from the bipotential primordial germ cells. Signaling mechanisms in the fetal testis and ovary determine whether germ cells enter the male or female developmental pathway, respectively. These signaling processes underpin an important phase of germ cell development, disruption of which can lead to failed germ cell function resulting in infertility or the formation of germ cell tumours.

**Findings:**

In this study we have developed a small molecule screening protocol combined with flow cytometry to identify signaling pathways that direct male-specific development of germ cells. Here we provide a detailed method for this screening protocol, which we have used to identify signaling pathways important for male germ cell development.

**Conclusion:**

This method will be of particular use in screening inhibitors of signaling pathways, endocrine disruptors or other chemicals for their ability to disrupt testis and germ cell development, thereby providing insight into testicular dysgenesis and factors underlying poor male reproductive health.

## Background

Spermatogenesis and oogenesis are founded on the development of the male and female germ cell lineages in the developing fetus. In mammals, primordial germ cells are specified from the pluripotent epiblast in the pre-gastrulation embryo [1]. The primordial germ cells migrate through the embryo and populate the developing gonads at approximately embryonic day (E) 10.5. At this stage, germ cells express key regulators of pluripotency and can readily establish pluripotent embryonic germ cell lines *in vitro,* reflecting their ability to retain germ line totipotency [2-5]. After entering the developing testes or ovary the germ cells differentiate down the spermatogenic or oogenic pathways in response to their respective environments [6-9]. The molecular pathways directing male and female germ line development are poorly understood, even though these processes are crucial for later fertility and for preventing germ cell tumours.

Testis development is initiated with the expression of Sex Region Y chromosome (*Sry)*, which activates *Sry* box gene 9 (*Sox9*), thereby promoting specification and proliferation of Sertoli cells and a cascade of events culminating in testis differentiation [10-12]. Sertoli cells proliferate and form testis cords, which enclose the fetal germ cells and define the interstitial space in which Leydig cell differentiation occurs [10,13]. These somatic cell types promote testis development and differentiation of the male germ line.

At the onset of testis or ovary development the germ cells can enter the male or female developmental pathway, regardless of whether they are genetically male (XY) or female (XX). The earliest indication of male and female germ cell development is their entry into mitotic arrest or meiosis, which occurs from E12.5 and E13.5, respectively [8]. Experimental evidence collected over the last three decades demonstrate that male and female germ cells are responsive to signals from the gonadal environment and this signaling leads to sex specification by E12.5 and E13.5, respectively [6,8,9,14].

The signaling mechanisms leading to male germ cell development and regulating testis development are not fully understood. However, germ cell mitotic arrest occurs in 48 hours from approximately E12.5, depending on the mouse strain [15,16]. Mitotic arrest of male germ cells involves activation of a number of G1-S phase check-point controlling proteins, including Retinblastoma and p27^KIP1^[15,17]. Male germ cell differentiation also involves the repression of the core pluripotency genes *Oct4*, *Sox2* and *Nanog*, which are maintained in germ cell derived teratomas [4,5,18-20].

We have previously analysed male germ cell development using flow cytometry [15,16,21]. In this study we aimed to develop an *ex-vivo* screening protocol for identifying signaling pathways involved in male germ cell development with the expectation that disrupting these signaling processes would block male germ cell mitotic arrest and differentiation, without causing sex-reversal. We isolated E12.5 fetal testes after male sex determination had occurred and testis cords had formed, but before germ cells had entered mitotic arrest [15,16]. These fetal testes were cultured with a range of specific small molecule chemical inhibitors and germ cell mitotic arrest was monitored using a flow cytometric assay. Here we provide a detailed account of this protocol and its application in screening small molecule inhibitors for their ability to disrupt mouse fetal germ cell or gonad development. This system provides an effective medium throughput, *ex-vivo* model for identifying small molecules or chemicals, such as endocrine disruptors, that inhibit germ cell mitotic arrest, reflecting compromised differentiation of the fetal germ cells, and potentially gonad development.

## Findings

### Materials required

•Click-iT-EdU 647 Flow Cytometry kit (Molecular Probes/Life Technologies C10424)

•Bovine Serum Albumen (BSA)

•Donkey serum

•Propidium Iodide 5 mg/ml in water

•RNaseA 20 mg/ml

•Phosphate Buffered Saline (PBS)

•Rabbit-anti-Mouse Vasa Homologue (MVH) antibody (AbCam Ab13840). It is advisable to perform a trial run to check your antibody stock and dilution prior to undertaking a larger experiment.

•Alexa fluor donkey-anti-rabbit 488 nm secondary antibody (Molecular Probes/Life Technologies; A21206)

•Cell culture medium

•Inhibitors reconstituted to appropriate concentration allowing at least 1 in 1000 dilution if in DMSO. We start with a concentration 10 × the IC50 for each inhibitor, or as otherwise recommended by the manufacturer. Do not use DMSO in cultures at less than 1 in 1000 dilution as it will affect the culture.

•Organ culture filters: 25 mm polycarbonate, polyvinyl pryolidine free (PVPF) filters, 12.0 micron pore size (GE Water and Process Technologies Catalogue number K12SH02500)

•Organ culture dishes (Nunc 353037) containing 1500 μl organ culture media

•Laminar flow hood, dissecting microscope and instruments

•Timed mated pregnant female mice or appropriate strain or line. We routinely use *Oct4*-GFP transgenic mice as these allow visualisation of the germ cells.

•Flow cytometer, micro-centrifuge, shaking heating block/water bath, cell culture incubator, 1.5 ml micro-centrifuge tubes etc.

### Culture medium

DMEM/F12 with glutamax: (Gibco/Life Technologies 10565)

10% Fetal Calf Serum: (or serum replacement; Gibco 10828)

15 mM Hepes: (1M stock; Gibco 15630)

1 × NEAA: (100× stock; Gibco 11140)

1 mg/ml N-acetyl-cysteine: (100× stock 100 mg/ml in water; Sigma A9165)

55 uM β-mercapto-ethanol: (1000× stock; Gibco 21985)

1 × Pen/Strep: (100× stock; Gibco 15070)

### Mice and tissue collection

All embryos used in the following protocols were derived from OG2 (*Oct4*-GFP) transgenic male × CD1 female matings. Mice were housed on a twelve-hour light/dark cycle. Mating was detected by the presence of a vaginal plug in the morning and recorded as E0.5. Embryos were sexed and staged according to morphological criteria. All animal procedures were carried out under approval of Monash University Animal Ethics or Murdoch Children’s Research Institute Animal Ethics Committees.

### Screening germ cell development by flow cytometric analysis

Initially in a series of single pass experiments (n=1, 4 gonads per culture) we screened 18 small molecule inhibitors for their ability to disrupt male germ cell development in fetal mouse testes. Testes were isolated from E12.5 *Oct4*-GFP positive mouse fetuses and cultured for 72 hours in the presence of inhibitor or vehicle control (e.g. dimethyl sulphoxide; DMSO) using a culture system we have previously validated [22]. In this screen the inhibitors used targeted a range of common signaling pathways, including those mediated by (or through) platelet derived growth factor (PDGF), glycogen synthase kinase 3 beta (GSK3β), mitogen activated protein kinase (MAPK), phosphatidylinositol 3-kinase (PI3K), janus kinase (JAK), vascular endothelial growth factor (VEGF) and ACTIVIN/NODAL/TGFβ. At the end of the culture period the gonads were labeled for two hours with 5-ethynyl-2′deoxyuridine (EdU), which marks cells progressing through S-phase.

**Figure 1:**
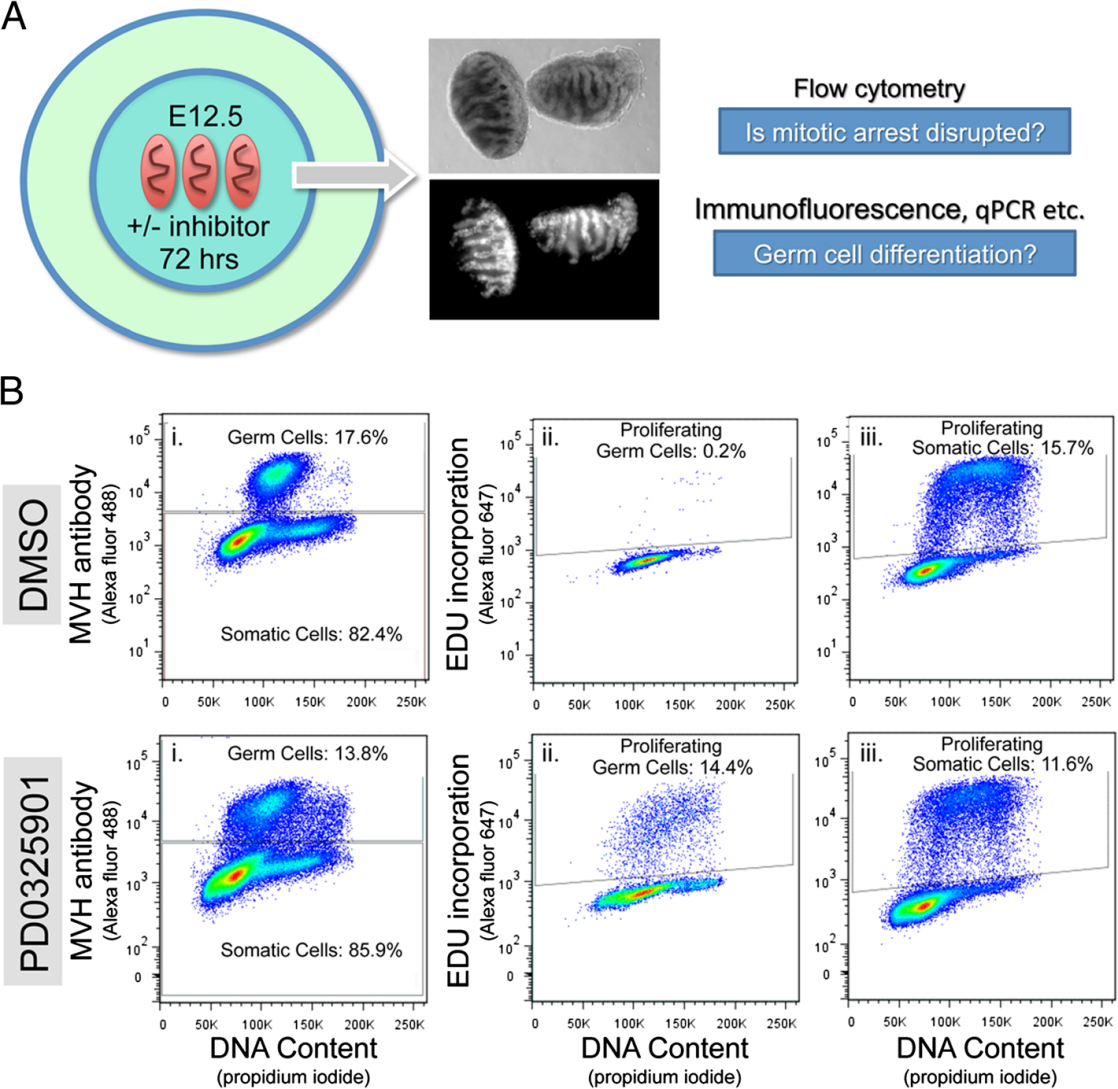
**Screening germ cell development using organ culture and flow cytometry. A**. Organ culture system showing brightfield (upper middle) and fluorescence (lower middle) images of E12.5 testes cultured for 72 hours. **B**. Flow cytometric output for (i) germ and somatic cells separated on the basis of MVH staining and proliferation of (ii) germ cells and (iii) somatic cells assessed by EdU staining in DMSO and PD0325901 treated gonad samples cultured for 72 hours.

After 72 hours of culture we examined gonadal phenotype in whole mount to identify overall effects of the inhibitors on gonad development. Testis cord morphology was viewed under phase contrast microscopy, while germ cells were viewed *in-situ* using GFP fluorescence, which is driven by germ cell specific expression of the *Oct4*-GFP transgene (Figure 1A). The gonads were then dissociated and subject to flow cytometric assessment of germ cell mitotic arrest, used in this context as a marker of male germ cell differentiation (Figure 1B) [15,16].

Flow cytometric analysis of the gonads treated in these cultures revealed that the two most effective inhibitors of male germ cell mitotic arrest were SB431542 and PD0325901, which inhibit the ACTIVIN/TGFβ/NODAL receptors ALK4, ALK5 and ALK7, and MEK1/2 signaling, respectively. Treatment with SB431542 or PD0325901 resulted in 14.5% and 13.5% of the germ cell population incorporating 5′-ethynyl-2′-deoxyuridine (EdU) (i.e. transiting S-phase) compared with 3.2% in DMSO (control) treated gonads. Detailed analyses of the impacts of SB431542 on male germ cell development has been reported in detail elsewhere [22] (D. Miles, S. Wakeling and P. Western unpublished data). Here we provide a step-by-step protocol for screening similar chemical inhibitors and other reagents for their ability to disrupt male germ cell development.

### Protocol

This protocol is based on Click-iT Edu chemistry (Invitrogen). Germ cells are separated from gonadal somatic cells using an antibody specific for Mouse Vasa Homologue (MVH) (AbCam Ab13840), detected using an alexa fluor donkey-anti-rabbit 488 nm secondary antibody, but both the primary and secondary antibody can be varied depending on your target cell (eg anti-SOX9 can be used to isolate Sertoli cells and anti-FOXL2 or anti-GATA4 can be used for female somatic cells), flow cytometer and preference for secondary detection. This protocol is written for analysis of germ cells in the fetal testis, but can also be applied to fetal ovary.

1. Organ culture

1.1: Prepare 2.0 ml culture medium containing either the vehicle control (e.g. typically DMSO or PBS, depending on the solubility of the inhibitor), or the inhibitor diluted at a 10 fold higher concentration than the IC-50 as declared by the manufacturer. For some compounds this may not be known and must be determined empirically. Pipette 1.5 ml culture medium into the inner well of a Nunc organ culture dish and place an organ culture filter over the well, overlapping the well edges and covering the medium. Culture medium should wet, but not cover membrane. Fill outer well of the organ culture dish with 4 ml sterile PBS to maintain humidity and store in the cell culture incubator until further use.

1.1: In an aseptic environment (eg laminar flow hood) dissect the required number of fetal gonads from E12.5 mouse embryos in PBS and separate gonads from mesonephros. You can use either gonad only or gonad with mesonephros attached for the culture. In our experience germ cells in E12.5 fetal testis/mesonephros and E12.5 testis only samples enter mitotic arrest with similar efficiency. Using testis only allows effects on both the germ cell and somatic compartments of the testis to be monitored simultaneously in the screening process.

1.1: Collect tissue for flow cytometry controls as outlined in Table 1.

1.1: Place 2–4 gonads onto each membrane (screening can be done with two gonads, but four provides better germ cell numbers at the end of the flow cytometric analysis). Culture gonads in a 5% CO2/air environment, at 37°C for 72 hours. Change medium daily to ensure activity of inhibitor is maintained.

1.1: Two hours before the end of the culture period add 1.5 μL 10 mM EdU to all samples except the EdU negative control and the MVH alexa fluor 488 positive control (optional) and the PI single colour control (optional) (Table 1). Incubate for two hours at 37°C.

1.1: After two hours of EdU treatment remove all culture medium and replace with PBS and remove gonads from the membrane by washing them off the membrane into the culture dish using a 1 ml pipette. Inspect and photograph under low power on an inverted fluorescence/bright field equipped microscope. Once you have the desired images collect gonads into a 1.5 ml micro-centrifuge tube for flow cytometry. We use maximum recovery tubes, but standard tubes can also be used.

2. Staining for flow cytometry

The following protocol requires around four hours depending on the number of samples.

2.1: Allow gonad and mesonephros samples to settle and wash once with PBS. Remove PBS and add 100 μL of 0.25% trypsin.

2.1: Incubate at 37°C for 5 mins, gently flicking tube approximately every 2 minutes or use a shaking heating block.

2.1: Transfer tubes to ice, add 500 μL of DMEM/10% FCS and dissociate cells by pipetting. Filter cells through cell filter (BD 352235) into a 1.5 ml micro-centrifuge tube, rinse original tube and filter with 200 μL extra DMEM/10% FCS. Collect cells at 3500 rpm (1000g) in a fixed angle bench-top centrifuge for 1 min. Rotate micro-centrifuge tube 180 degrees and spin again for 2 mins. Cells will form pellet at one side of tube base. Carefully remove supernatant from opposite pellet, leaving 10 ul behind.

2.1: Resuspend in 100 μL of fixative (4% Paraformaldehyde in PBS) for 15 mins at room temperature (RT). Collect cells at 5000 rpm (2500 g) 10 seconds at RT. Rotate micro-centrifuge tubes 180 degrees and spin again for 10 seconds.

2.1: Wash cells in 100 μL of permwash (supplied as part of the Click-iT-EdU 647 Flow Cytometry kit). Collect cells at 5000 rpm 10 seconds. Rotate tubes 180 degrees and spin again for 10 seconds. Repeat one more time. (Potential stopping point: Cells can be stored in permwash for at least one week if staining for MVH)

2.1: Spin down and then resuspend again in 50 μL permwash with 10% donkey serum (or appropriate species serum for secondary antibody). Incubate for 10–20 mins at RT.

2.1: Collect cells at 5000 rpm 10 seconds. Rotate tubes 180 degrees and spin again for 10 seconds. Resuspend in 25 μL permwash containing anti-MVH antibody diluted 1/500. Incubate for 30–45 mins at room temperature (RT).

2.1: Add 100 μL of permwash to each tube and spin at 5000 rpm 10 seconds. Rotate tubes 180 degrees and spin again for 10 seconds.

2.1: Wash cells in 100 μL of permwash. Collect cells at 5000 rpm 10 seconds. Rotate tubes 180 degrees and spin again for 10 seconds. Repeat one more time.

2.1: Resuspend in 25 μL permwash containing donkey anti-rabbit secondary antibody (donkey anti-rabbit 488 nm) diluted 1/300. Incubate for 30–45 mins at RT in the dark.

2.1: Add 100 μL of permwash to each tube and spin at 5000 rpm 10 seconds. Rotate tubes 180 degrees and spin again for 10 seconds.

2.1: During washing prepare the 1× Click-iT EdU reaction buffer additive by diluting 1:10 in MilliQ water and the Click-iT reaction cocktail according Table 2. The reaction cocktail should be added to the cells within 15 mins of preparation, so don’t prepare too early (volumes in table can be scaled according to sample size).

2.1: Wash cells in 100 μL of permwash. Collect cells at 5000 rpm 10 seconds. Rotate tubes 180 degrees and spin again for 10 seconds.

2.1: Add 200 μL of Click-iT reaction cocktail (Table 2) to each tube and mix well.

2.1: Incubate for 30 minutes at RT in the dark.

2.1: Collect cells at 5000 rpm 10 seconds. Rotate tubes 180 degrees and spin again for 10 seconds. Wash cells once in 100 μL of permwash.

2.1: Add 400 μL permwash containing 4 μL 5 mg/ml PI and 1.0 μL 20 mg/ml RNaseA.

3. Flow cytometric analysis

Perform flow cytometry using the following protocol. Once you know how to use the flow cytometer, you will require about 20 mins to set up gates and 3–5 mins per sample running time. Set up gates running samples on low speed, and analyse your samples on medium-high.

**Figure 2:**
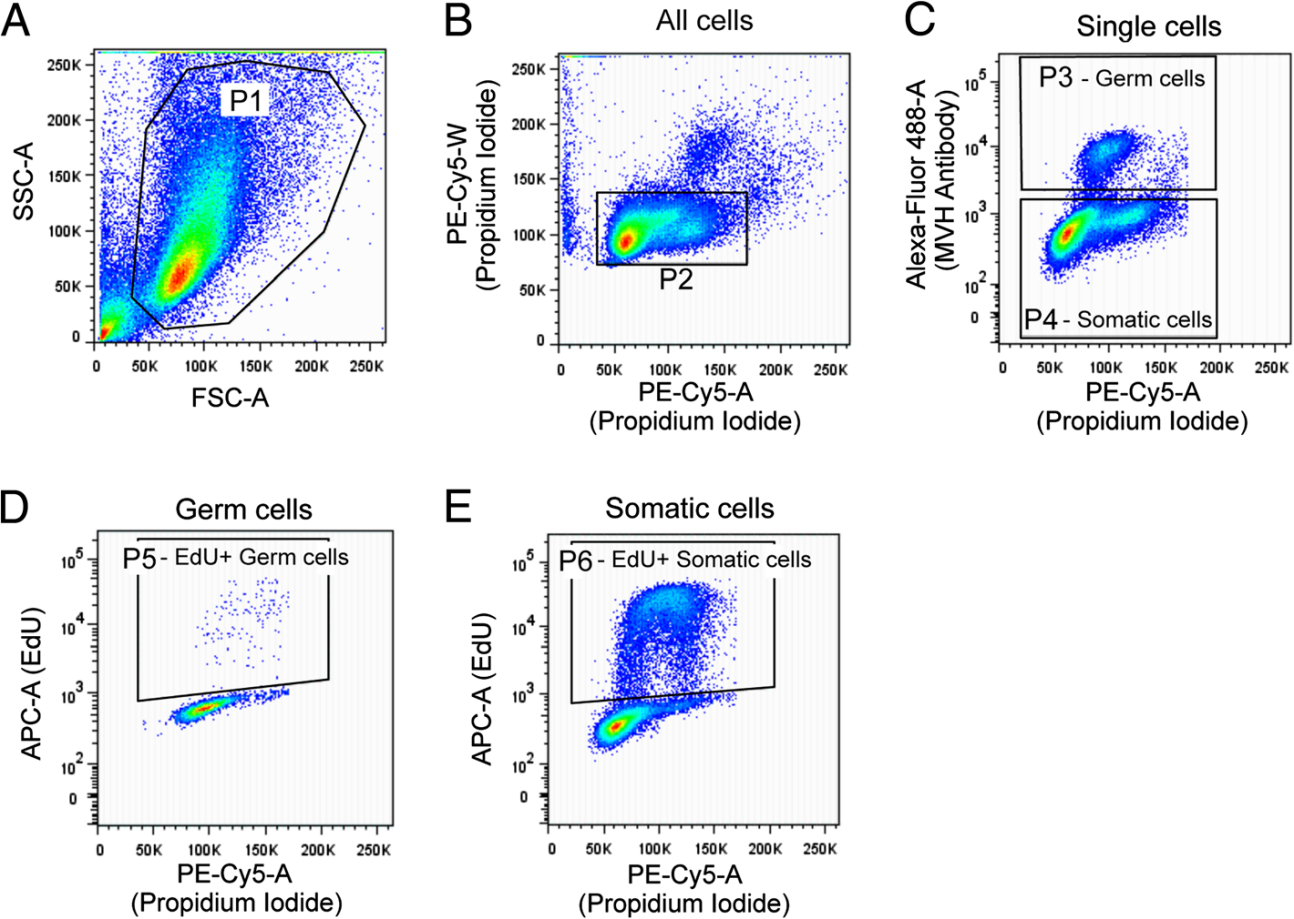
**Examples of gating strategy for flow cytometric analysis of fetal germ cells and gonadal somatic cells based on a PBS treated control sample. A**. Side vs forward scatter plot showing gate P1 around cells and excluding cell debris. **B**. Propidium iodide in the PE-Cy5 width vs propidium iodide in the PE-Cy5 area showing events from P1, with single cells gated within P2. **C**. MVH-alexa fluor 488 vs propidium iodide area showing events from P2, with germ cells (MVH positive) gated in P3 and somatic (MVH negative) cells gated in P4. **D**. EdU-Click-iT 647 in the APC-area channel vs propidium iodide area showing events from P3 (ie germ cells), with proliferating (EdU positive) cells gated in P5. **E**. EdU-Click-iT 647 in the APC-area channel vs propidium iodide area showing events from P4 (ie somatic cells), with proliferating (EdU positive) cells gated in P6.

3.1: Using the control sample 1 (Table 1) establish a side scatter vs forward scatter plot. Isolate cells from debris using gate P1 (Figure 2A). Display cells from P1 in a new scatter plot of PI width (linear) vs PI area (linear) (Figure 2B).

3.1: Using P1 as the parent population, establish a gate (P2) around the single cells as shown in Figure 2B. Set PI intensity so that the G1 population is located over 50 to100 and G2/M population is located over 100 to 200 fluorescence units (Figure 2B). This value is not critical, as long as sufficient separation is achieved using PI to model cell cycle in a third party software such as ModFit or FlowJo.

3.1: Showing only the P2 events and using P2 as the parent population, establish a scatter plot for alexa-flour 488 nm (log scale) vs PI (PE-Cy5) area (linear scale). Set 488 nm laser power so that the 488 nm negative somatic cells are below just 1000.

3.1: Change to control sample 2 (Table 1). You should see MVH positive germ cells in the 1000–10,000 range. Expect the staining intensity for MVH to be a little higher in males than females and higher at E15.5 than at E12.5. In an untreated E12.5 samples cultured for 72 hours most of the MVH positive cells should be located over the G1 PI value (ie over 50–100) with few scattered over S-G2/M (100–200) (Figure 2C).

3.1: Establish a gate (P3) around the MVH 488 nm positive cells, extending above the 488 negative cells and incorporating PI values approximately 50–300 to ensure all cells are included in treated samples to be analysed (Figure 2C). These are the germ cells. Establish a similar gate (P4) around the MVH 488 nm negative cells. These are the somatic cells.

3.1: To assess germ cell proliferation establish a scatter plot for EdU (APC) area 647 nm (log scale) vs PI (PE-Cy5) area (linear scale), showing only the P3 events. Set 647 nm laser power so that the 647 nm negative cells are below just 1000. Using P3 (germ cells) as the parent population, establish a gate (P5) around the 647 nm positive cells. P5 contains germ cells passing through S-phase in the two-hour culture period during which the gonads were exposed to EdU (Figure 2D).

3.1: To assess somatic cell proliferation establish another plot, similar to that established for germ cells in 3.6. Showing only the P4 events establish a scatter plot for EdU (APC) area 647 nm (log scale) vs PI (PE-Cy5) area (linear scale). Set 647 nm laser power so that the 647 nm negative cells are below just 1000. Using P4 (somatic cells) as the parent population, establish a gate around the EdU (APC) area 647 nm positive cells (Figure 2E). P6 should be essentially identical to the somatic cell P5 gate, but specific for the somatic cells. These are the somatic cells passing through S-phase in the two-hour culture period during which the gonads were exposed to EdU.

3.1: Using control sample 1 (Table 1) check your P3, P4, P5 and P6 gates to ensure they are correctly established.

3.1: Run samples

3.1: Analyse data accordingly. You can analyse the percentage of EdU positive germ cells and somatic cells in each sample. You can also perform cell cycle analysis based on PI staining for the germ cell and somatic cell populations using ModFit, FlowJo or similar programs. For example, in a comprehensive analysis using DMSO and the inhibitor SB431542 to treat gonads in culture, 3.2 ±0.6% (SEM, n=10 cultures) and 14.3 ±3.3 (SEM, n=10) (p<0.001) of the germ cells incorporated EdU, respectively. However, in the somatic cell population 15.9% ± 0.6 (SEM, n=10) and 12.3 ± 0.81% (SEM, n=10) of the DMSO and SB431542 treated cells incorporated EdU, respectively (p<0.001) [22].

**Table 1:** Flow cytometry controls

**Control sample**	**EdU**	**Stain**	**Purpose**
1. Mesonephroi × 4	No	• Click-iT 647	Negative sample for establishing gates in the 647 nm and 488 nm channels
		• MVH-alexa fluor 488	
		• Propidium iodide	
2. Gonads × 4	Yes	• Click-iT 647	Positive controls for setting the laser power in the 488 nm and 647 nm channels. Use females gonads for this if concentrating of male germ cell development and vice-versa if concentrating on female germ cell development (or a mix, if concentrating on both).
		• MVH-alexa fluor 488	
		• Propidium iodide	
3. Mesonephroi × 2 (optional)	Yes	• Click-iT 647 only	Single color control for staining compensation
4. Mesonephroi × 2 (optional)	No	• Propidium iodide only	Single color control for staining compensation
5. Gonads × 2 (optional)	No	• MVH-alexa fluor 488 only	Single color control for staining compensation

**Table 2:** EdU-Click-iT labeling mix

**Component**	**Volume / tube**
PBS	175 μL
CuSO_4_ (100 mM)	4 μL
647nm dye azide	1 μL
Reaction buffer additive	20 μL
Total volume	200 μL

## Discussion

Over recent decades male reproductive health has declined and it has been suggested that various chemicals or environmental toxins may be responsible through their ability to disrupt testis and germ-line development [23]. This is manifest in increased incidence of pluripotent germ cell tumours, which have their origin in poorly differentiated fetal germ cells. Male germ cell differentiation is initiated and directed by unknown signaling processes within the fetal testis, which results in strict regulation of germ cell proliferation and the repression of germ cell pluripotency. Although these signaling processes are poorly understood, disruption of male germ cell development at this stage can lead to pluripotent germ cell tumours [19,20].

A tractable and sensitive model that facilitates rapid screening of signaling inhibitors, environmental toxins (e.g. endocrine disruptors) or other reagents that result in compromised testis and/or germ cell development would be of significant benefit in male reproductive biology. We have combined our existing flow cytometric method with gonad organ culture to develop a drug screening protocol that facilitates identification of key signaling pathways in testis and male germ cell development. Our model provides a means to rapidly screen drugs that affect the early processes in testis and male germ cell development and allow the analysis of perturbed germ cell differentiation in a compromised testicular environment.

By using E12.5 rather than E11.5 testes this protocol provides a method for perturbing germ cell development after somatic sex determination, reducing the complicating factors introduced by sex-reversal. The flow cytometric assay allows robust detection of small changes in cell proliferation in small amounts of tissue (2–4 gonads are sufficient). As quality antibodies suitable for flow cytometry are commercially available and/or existing transgenic mouse models provide access to various testis specific cell types (i.e. germ cells, Sertoli cells, endothelial cells, Leydig cells) [24], this approach is applicable to screening many inhibitors, endocrine disruptors or other reagents that might impact testis or germ cell development.

To test the veracity of the screening approach we initially screened 20 inhibitors of common signaling pathways for their ability to disrupt male germ cell development in the early testis, soon after male sex determination. We identified an important role for ACTIVIN/NODAL/TGFβ signaling pathways in male germ cell development, which we analyzed in detail in a separate study [22]. We also identify negative affects on mitotic arrest of fetal male germ cells using the MEK1/2 (MAPK) inhibitor germ cell PD0325901 (S. Wakeling, D. Miles and P. Western, unpublished data) and are now performing a more detailed analysis of this pathway.

Following identification of drugs or chemicals that exhibit activity in this screening protocol, further detailed analyses, including immunofluorescence, qRTPCR and cell biological assays can be performed to establish the mechanisms through which the inhibitor is active. Given appropriate antibodies for flow cytometry, a similar approach could be applied to other stages of gonad development and may allow identification of drugs, chemicals or toxins leading to reduced fertility and germ cell function. We have applied this screening approach to *ex-vivo* gonad cultures in mice because germ cell development is best characterized in this species and *ex-vivo* screening facilitates a higher throughput system. However, the same approach can be applied to mice using *in-vivo* treatment, or a similar approach could be used in other species.

We have developed a robust protocol facilitating medium throughput screening of chemical inhibitors that can be used to identify signaling pathways involved in male germ cell development. This model provides an accessible system in which environmental and developmental processes influencing testis and male germ cell development can be manipulated and will provide important insights into the processes underlying testicular dysgenesis and the early stages of germ cell tumour formation.

## Competing interests

The authors have no competing interest to declare.

## Authors’ contributions

SIW: experimental design, performed and analysed experiments. DCM experimental design, performed and analysed experiments, wrote the manuscript. PSW experimental design, performed and analysed experiments, wrote the manuscript, corresponding author. SIW and DCM share equal authorship. All authors read and approved the final manuscript.
